# Myopia progression from wearing first glasses to adult age: the DREAM Study

**DOI:** 10.1136/bjophthalmol-2020-316234

**Published:** 2021-01-25

**Authors:** Jan Roelof Polling, Caroline Klaver, Jan Willem Tideman

**Affiliations:** 1 Ophthalmology, Erasmus MC, Rotterdam, Zuid-Holland, The Netherlands; 2 Orthopics & Optometry, Hogeschool Utrecht, Utrecht, The Netherlands; 3 Ophthalmology and Epidemiology, Erasmus MC, Rotterdam, Zuid-Holland, The Netherlands; 4 Ophthalmology, Radboudumc, Nijmegen, Gelderland, The Netherlands

**Keywords:** epidemiology, public health

## Abstract

**Purpose:**

Data on myopia progression during its entire course are scarce. The aim of this study is to investigate myopia progression in Europeans as a function of age and degree of myopia from first prescription to final refractive error.

**Methods:**

The Drentse Refractive Error and Myopia Study assessed data from a branch of opticians in the Netherlands from 1985 onwards in a retrospective study. First pair of glasses prescribed was defined as a spherical equivalent of refraction (SER) ≤−0.5 D to ≥−3.0 D. Subjects with prescriptions at an interval of at least 1 year were included in the analysis.

**Results:**

A total of 2555 persons (57.3% female) met the inclusion criteria. Those with first prescription before the age of 10 years showed the strongest progression (−0.50 D; IQR: −0.75 to −0.19) and a significantly (p<0.001) more negative median final SER (−4.48 D; IQR: −5.37 to −3.42). All children who developed SER ≤−3 D at 10 years were highly myopic (SER ≤−6D) as adults, children who had SER between −1.5 D and −3 D at 10 years had 46.0% risk of high myopia, and children with SER between −0.5 D and −1.5 D had 32.6% risk of high myopia. Myopia progression diminished with age; all refractive categories stabilised after age 15 years except for SER ≤−5 D who progressed up to −0.25 D annually until age 21 years.

**Conclusion:**

Our trajectories of the natural course of myopia progression may serve as a guide for myopia management in European children. SER at 10 years is an important prognostic indicator and will help determine treatment intensity.

## Introduction

The current worldwide increase in myopia prevalence is leading to a growing public health burden, as the more high levels of myopia (≤−6 D) can lead to blinding complications such as myopic macular degeneration, retinal detachment, glaucoma and choroidal neovascularisation.^1–3^ Risk factors for high myopia at adult age are a young age of onset and a fast progression rate during childhood.^4–6^

Myopia onset occurs typically during childhood, teenage years or adolescence.^4 7^ The average age of myopia onset varies among gender, ethnicities and presence of parental myopia.^8^ Other established risk factors for myopia are more intense education, less time outdoors and increased near work that appear to coincide with an earlier onset.^9–12^ The strongest progression of eye growth is observed in early childhood, while stabilisation may not occur until late adolescence.^13 14^ Around 90% of all myopic individuals appear to be stable at the age of 21 years, and nearly all by the age of 24 years.^8^

Most existing data on myopia progression have been provided by controlled myopia intervention studies, which have a short follow-up period, limited numbers or are based on imputed data.^8 15 16^ Longitudinal studies in Europeans with 10-year follow-up are available, but time interval between the onset of myopia and final refractive error might be longer. This limits robust insights into the association between age of onset and final refractive error.^17^ The aim of this study is to describe myopia growth trajectories and the association between the first myopic prescription and final refractive error in a cohort of European children.

## Methods

### Study population

The Drentse Refractive Error And Myopia (DREAM) Study population comprised of subjects who bought their glasses from 1 of the 14 dispensing opticians from a chain of stores belonging to 1 family. The stores were located in the north of the Netherlands including the provinces Overijssel, Friesland, Groningen and Drenthe. The area has 1.7 million inhabitants and is classified as a non-urban area with 37% of the people living in an urban environment.^18^ Ethnicity was an unknown variable in this study; however, according to the open source Statistics Netherlands’ database, personsin the region with a non-western background was approximately 3% in 1980 to 5% in 2015.^18^ Records of eyeglass orders were stored digitally since 1985, and all data gathered since that time up to 2015 entered the current analysis. Eligibility criteria were at least two orders of myopic eyeglasses with an interval of 1 year or more until the age of 25 years. Final degree of myopia was obtained from a visit between 22 and 25 years of age. Subjects were born between 1962 and 1997; follow-up time ranged from 1 to 22 years with a mean of 5.82 years (SD 4.1). Data were completely anonymised by the dispensing opticians and in full compliance with the European General Data Protection Regulation.

### Refractive error and myopia

Assessment of refractive error was done by multiple eye care providers, however, compatible with Dutch health guidelines, refractive error was determined by an orthoptist or an ophthalmologist under cycloplegia up to 12 years of age, and was performed by a qualified optician at older ages. Spherical equivalent of refraction (SER) was calculated as an average sphere +½ cylinder for both eyes. Myopia was defined as SER of −0.50 D or worse of the prescribed glasses and high myopia was defined as ≤−6.00 D. Contact lens data were used if subjects moved from glasses to contact lenses. The back vertex formula in reversed order was used to calculate the contact lens prescription into those of glasses Fg=Fc/(1+xFc) in which Fg is the glasses prescription in diopters, Fc contact lenses prescription and x the vertex distance in metres (0.0125).

### Statistical analysis

First purchases of myopic eye glasses with refractive error up to −3 D were eligible for the primary analysis; first purchases with more severe myopia were only eligible for myopia progression analyses. Data were presented as medians and IQRs, the percentiles or numbers and percentages. SER and progression rates showed a non-normal distribution, and a non-parametric test was used. Differences in progression between spherical equivalent groups (−1 D to −2 D; −2 D to −3 D; −3 D to −4 D; −4 D to −5 D; −5 D to −6 D; −6 D to −7 D) at baseline were compared using Kruskal-Wallis test; differences between female and male progression using Mann-Whitney U test. The association of SER progression at different age intervals in the same children was determined using Spearman’s correlation. The mean myopic SER and the percentiles were calculated per age group (<10 years n=253; 10–12 y/a n=562; 13–15 y/a n=729; 16–18 y/a n=882; 19–21 y/a n=1270). The progression in SER from one age group to the subsequent age group was calculated, as were annual progression rates by the ratio between SER progression and time between visits. For the distribution of myopia progression per age category, we calculated the 5th, 10th, 25th, 50th, 75th, 90th and 95th percentile values of myopic SER as annual progression. The cumulative risk of incident high myopia (ie, an SER of −6.0 D or more) was estimated by Kaplan-Meier product limit analysis stratified for first myopic prescription and SER categories. P values below 0.05 were considered statistically significant for all statistical tests. All statistical tests were performed by using IBM SPSS Statistics for Windows, V.25.0.

## Results

A total of 2555 (57.3% female) subjects were eligible for the progression analyses; 946 (37.0%, 59.2% female) had a first myopic prescription (SER between −0.5 D and −3 D) and refraction at adult age (22+ years) (online supplemental table 1). Median refractive error at adult age for the complete cohort was −2.50 D (IQR; −4.01 to −1.5), the proportion of high myopia was 8.9% (n=113).

10.1136/bjophthalmol-2020-316234.supp1 Supplementary data

**Figure 1:**
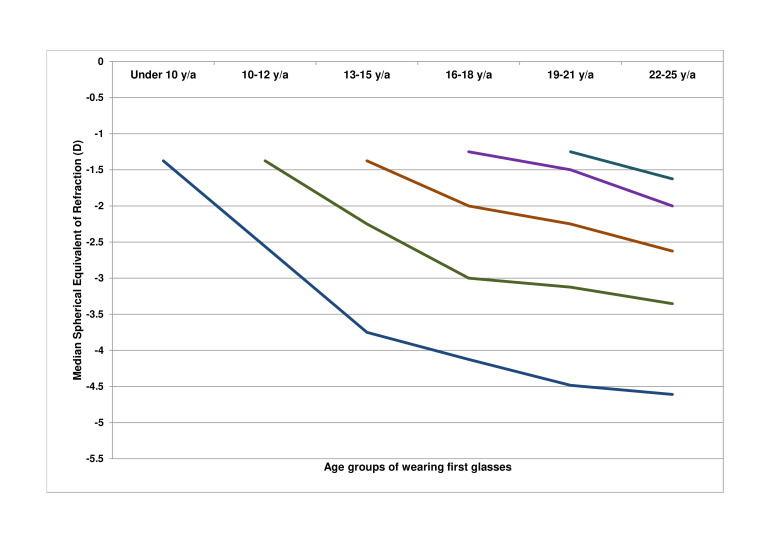
Median spherical equivalent of refraction in diopters in children from first prescription of myopia and adult myopia obtained at the age of 22–25.

**Figure 2:**
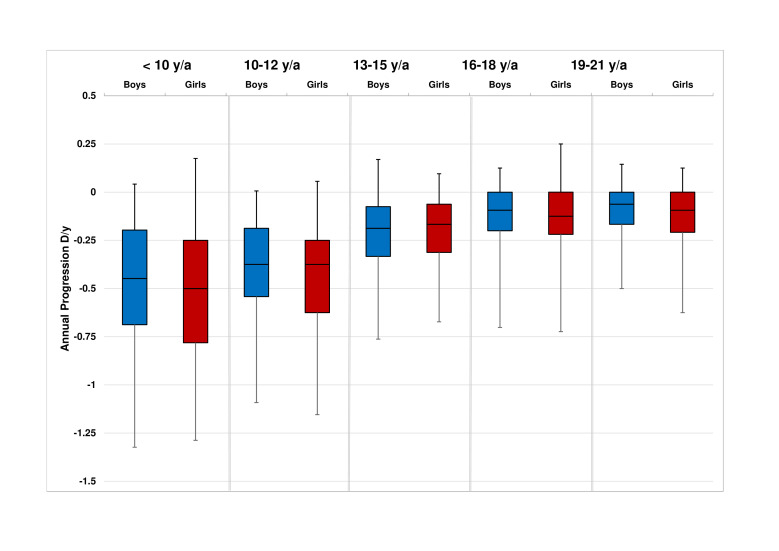
Boxplots of median annual progression of spherical equivalent of refraction in diopters for boys (blue) and girls (red) per age group. Lower and upper box boundaries 25th and 75th percentiles and lower and upper error lines 2.5th and 97.5th percentiles. Tested with non-parametric Mann-Whitney U test.

**Figure 3:**
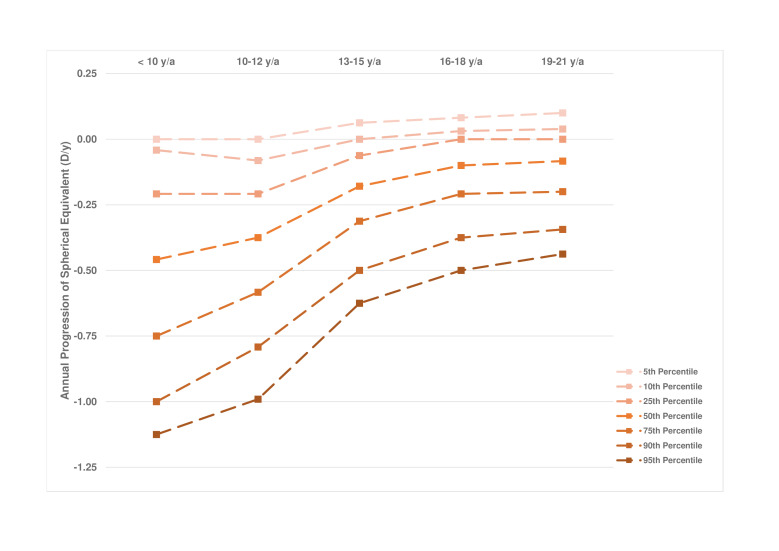
Progression curves in percentiles representing annual progression rate of spherical equivalent in diopters as a function of age for European myopic subjects. Percentiles were calculated per age group and are connected by dashed lines.

**Figure 4:**
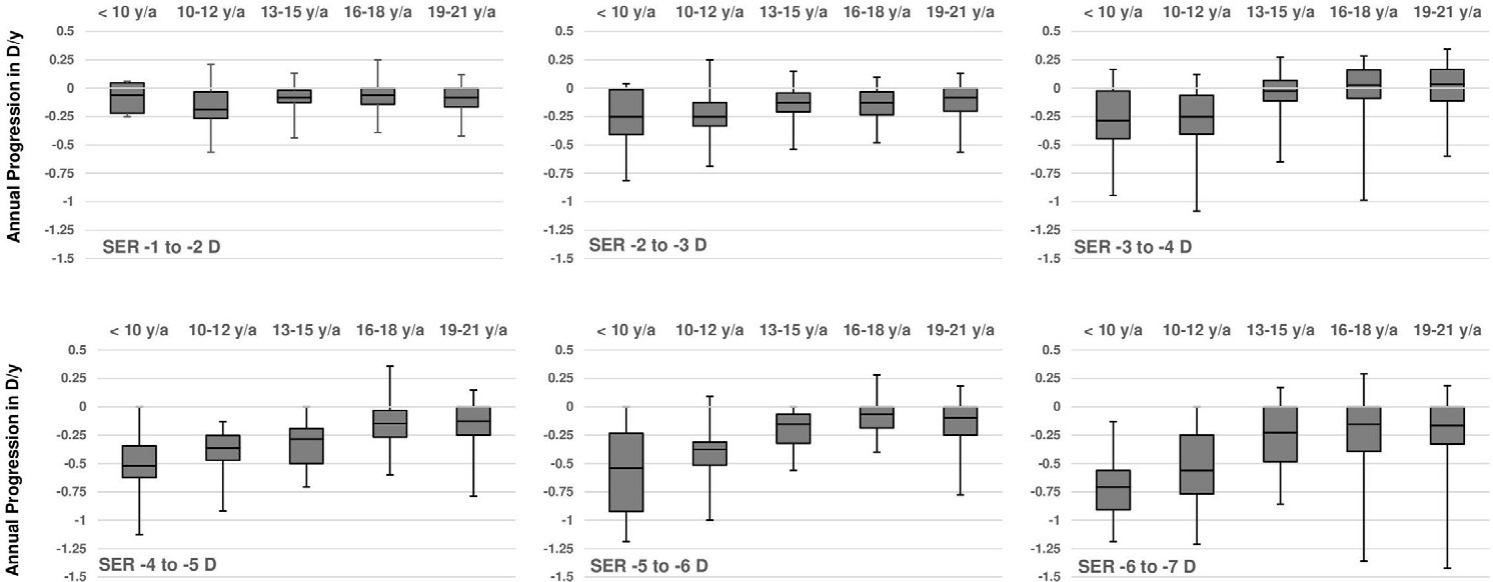
Boxplots of median annual progression in diopter spherical equivalent per adult degree of myopia category obtained at the age of 22–25. Lower and upper box boundaries 25th and 75th percentiles and lower and upper error lines 2.5th and 97.5th percentiles. SER, spherical equivalent of refraction.

Figure 1 shows the progression of SER per age of onset category (n=946). Earlier first myopic prescription was significantly associated with a higher degree of myopia (p<0.001) at adult age. The median annual progression of SER decreased with age; this was −0.50 D (IQR: −0.75 to −0.19) in ages up to 10 years; −0.38 D (IQR: −0.63 to −0.19) at ages 10–12 years; −0.19 D (IQR: −0.34 to −0.06) ages 13–15 years; −0.09 D (IQR: −0.21 to 0) at ages 16–18 years; and −0.08 D (IQR: −0.21 to 0) at ages 19–21 years. Female subjects showed a significantly stronger progression in only one age category: 19–21 years, −0.09 D females versus −0.06 D males (p=0.01; figure 2). Figure 3 shows the annual progression of SER per age category. The annual progression is much greater for those with early onset myopia ≤12 years compared with over 12 years. Until 12 years, median progression was more than −0.25 D/year; beyond 16 years, only the 90th and 95th percentile progressed more than −0.25 D/year. Plots for the median annual progression per age category stratified for adult SER are shown in figure 4. Subjects with high myopia at adult age had progressed with −0.71 D/year (IQR: −0.91 to −0.56) up to age 10 (figure 4fF); milder myopes at adult age had progressed at a lower rate in the first decade (figure 4A–E).

**Figure 5:**
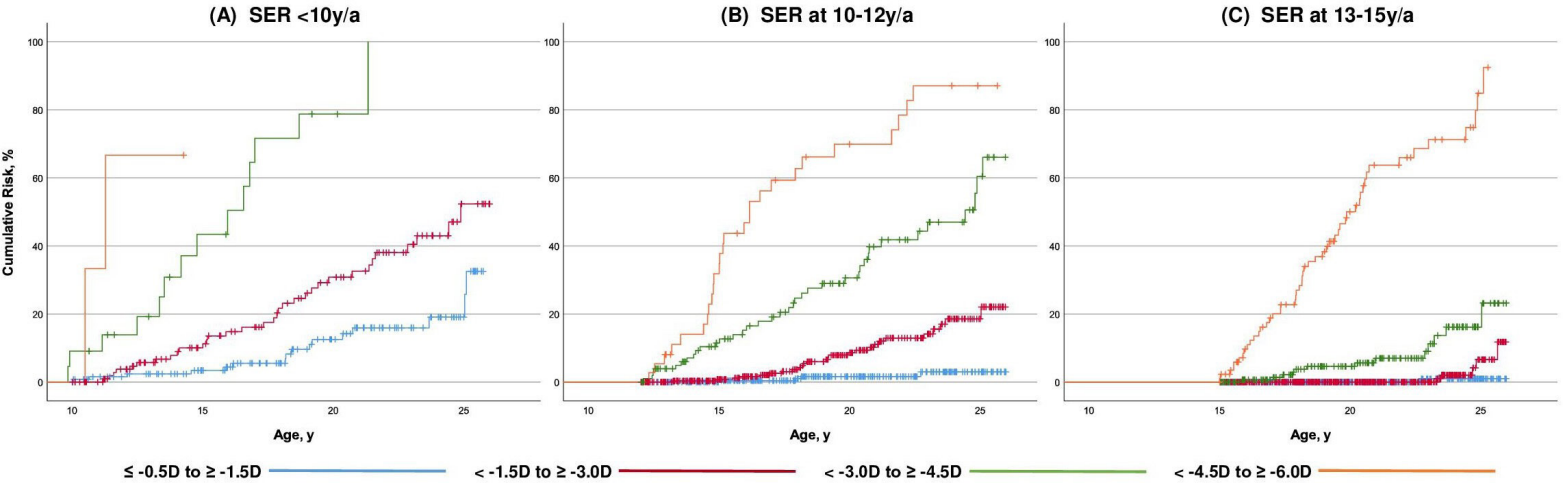
(A–C) Cumulative risk of high myopia (≤−6 D) according to spherical equivalent of refraction in diopters category by age. (A) For subjects with myopia onset younger than 10 years of age. (B) For subjects with myopia onset 10–12 years of age. (C) For subjects with a myopia onset 13–15 years of age. SER, spherical equivalent of refraction.

We estimated the risk of high myopia as a function of refractive error in age categories (figure 5, time to event curve, online supplemental table 2). All subjects with SER −3 D or worse in childhood up to 10 years developed high myopia. Those with SER −4.5 D to −6 D at age 10 years developed high myopia at 11.2 y/a (95% CI, 10.0 to 12.5), those with −3 D to −4.5 D at 10 years did so at 16.0 years (95% CI, 12.9 to 19.0). Remarkably, those with SER −0.5 D to −1.5 D and −1.5 D to −3.0 D up to 10 years still had, respectively, 32.6% and 46.0% risk to develop high myopia by age 25 years; those with SER −0.5 D to −1.5 D and −1.5 D to −3.0 D at 10–12 years had only 3.0% and 18.2% risk. Those who had SER −0.5 D to −1.5 D at later ages had virtually no risk of high myopia. However, those who had moderate myopia, SER −1.5 D to −3.0 D and −3 D to −4.5 D, at age 15 years still had 11.8% and 23.2% risk of developing high myopia. Correlation between progression at age <10 years and at 10–12 years was R=0.36; between 10–12 years and 13–15 years R=0.33; between 16–18 years and 19–21 years R=0.23 (all p≤0.01). Correlation between progression at 13–15 years and 16–18 years was R=0.13 (p=0.02).

10.1136/bjophthalmol-2020-316234.supp2 Supplementary data

## Discussion

This study describes myopia progression in 2555 European children who received glasses during childhood or teenage years and who were followed until age 25 years. The SER at adult age ranged from −0.5 D to −12.75 D, with a median of −3.00 D. Of those who developed high myopia, 60% had a first pair of glasses before age 10 years. Children who developed −3 D or worse in the first decade all developed high myopia. High myopes at adult age had been faster progressors during the entire youth, those with lower refractive errors virtually ceased progression after age 15 years.

Many clinics all over the world are offering myopia control using various strategies. Good myopia management requires insight into the natural course of myopia progression, as the goal is to slow down the rate. Clinical trials have attempted to provide these data by control groups, but the relatively short duration of these studies have hampered long-term predictions. Our study is unique as it studies myopia progression until age 25 years in a very large Dutch cohort. The cohort consisted of individuals who had bought their glasses at a branch of dispensing opticians from a family business, with a loyal clientele and a collective registration system. Progression rates in this cohort were in line with other European studies, suggesting that our results are generalisable to the European population at large.^8 17 19–21^

Potential limitations of our study should also be mentioned. The design was retrospective and included persons who developed myopia in the time period 1980–2000. Children growing up then may not be representative of children of today, who are likely to perform even more near work and spend less time outdoors. Participants were from an area with a relative low population density; only 37% lived in an urban environment.^18^ Nevertheless, this did not lead to lower progression rates than other studies in European children. Important risk factors such as outdoor exposure and near activities were not assessed in the study. This was a limitation because of the retrospective study design and could explain why children with mild myopia at age 10 still developed high myopia. Unfortunately, this cannot be explained by this study due to the lack of data on these and other risk factors for myopia progression. Another drawback is the classification of first prescription of ≤−0.5 D to −3.0 D instead of a variable onset of myopia which may have led to misclassification of persons to an older group.

Persons with a first myopic prescription before the age of 10 years developed a median SER of −4.48 D (IQR: −5.37 to −3.42) at adult age. In the American Correction of Myopia Evaluation Trial (COMET) carried out during the turn of the century, white children with a myopia onset at 6–11 years showed mean SER of −5.04 D (SD 0.14) at stabilisation.^8^ Our median annual progression in children younger than 10 years (SER −0.45 D; IQR: −0.69 to −0.20) and from 10 to 12 (−0.38 D; IQR: −0.19 to −0.54) corresponded well with the mean 3-year progression rate in the 8–12 year control group of the MiSight Lenses study (−1.24 D, SD 0.61 in 3 years), with the 7-year-old participants of an Australian cohort (−0.41 D) but was slightly more than the 3-year progression rate in the 6–7 to 9–10 year old white European children in the Northern Ireland Childhood Errors of Refraction (NICER) Study (−1.14 D, 95% CI, −3.13 to −0.63).^19 20 22^ Our rate in children aged 13–15 years (−0.19 D, IQR: −0.33 to −0.08) was slightly more than the mean 3-year progression rate in children from the NICER Study (−0.33 D, 95% CI, −1.63 to 0.63) but less than the annual rate of 13-year-old Australians (−0.31 D), though 47.3% of these children was of non-western background.^20 22^ Our rate appeared somewhat lower than the progression in 6–15-year-old children of the 2-year low dose atropine study from Los Angeles (−1.2 D; SD 0.7 in 2 years), but this retrospective study included mainly children from Asian ethnicity.^21^ Other Asian studies also reported higher rates (−0.8 D/year).^23^ Progression of myopia decelerated with age in our study to −0.05 (IQR: −0.13 to 0.0) in those aged 19–21 years, which was slightly less compared with the progression found in the mean annual progression by the NICER Study (−0.09, 95% CI, −0.51 to +0.19) in children 12–20 years.^24^ Higher degrees of adult myopia showed faster progression, especially before the age of 13 (figure 4). These age patterns confirm observations from others who also found the steepest progression patterns in the youngest age group.^8 17 20 25–27^

High myopia is clinically the most significant outcome of myopia. Our time-to-event curves provide insight for development of this refractive error category as a function of age (figure 5). All persons with SER −3 D at 10 years developed high myopia by adult age. We think this degree of refractive error developed in the first decade can serve as an indicator for professionals to maximise myopia control and lifestyle advice to reduce final refractive error. Unfortunately, lower refractive errors at age 10 did not exclude development of high myopia; hence, all children with a first myopic prescription below 10 years of age should be followed with care.

Similar to the children in the COMET Trial, gender was not associated with the final degree of myopia. Asian studies did find predilection for females, girls had both higher mean SER and stronger progression. Although we observed a slight gender difference in progression rate in one age category, this difference was minimum and did not exceed −0.03 D/year.^28^ Lifestyle seems to be a likely explanation to the findings in Asian girls.

In conclusion, our results provide myopic refractive error trajectories during the entire youth for Europeans and present the risk of high myopia as a function of age and refractive error in childhood. With its practical simplicity, the DREAM study can be used to evaluate myopia progression in white children and may serve as a guide for treatment outcomes in myopia control programmes.

## Data Availability

All data relevant to the study are included in the article or uploaded as supplementary information. The data that support the findings of this study were made available by Greving & Greving opticians. Restrictions apply to the availability of these data, which were used under license for this study. The authors confirm that the data supporting the findings of this study are available within the article and its supplementary materials.
