# Supplementary material for: Myopia progression from wearing first glasses to adult age: the DREAM Study
Source: Br J Ophthalmol. 2021 Jan 25;106(6):820–4. doi: 10.1136/bjophthalmol-2020-316234 (PMC9132855; doi:10.1136/bjophthalmol-2020-316234)
Supplement: Supplementary data [file bjophthalmol-2020-316234supp001.pdf]

Supplementary Table 1: Number of subjects in the analysis

**Total sample N = 2555***		
	**Progression**	
	Under 10 years – 10-12 years	N = 253
	10–12 – 13–15 years	N = 562
	13-15 – 16–18 years	N = 729
	16-18 – 19-21 years	N = 882
	19-21 – 22-25 years	N = 1270
	**First myopic prescription and measurement at 22-25 years**	**N = 946**
	Under 10 years – 22-25 years	N = 92
	10-12 – 22-25 years	N = 115
	13-15 – 22-25 years	N = 133
	16-18 – 22-25 years	N = 117
	19-21 – 22-25 years	N = 489
	**Cumulative risk high myopia**	
	Under 10 years	N = 279
	10-12 years	N = 751
	13-15 years	N = 1083

** Two orders of myopic eyeglasses with an interval of one year or more until the age of 25 years*
