# Supplementary material for: Myopia progression from wearing first glasses to adult age: the DREAM Study
Source: Br J Ophthalmol. 2021 Jan 25;106(6):820–4. doi: 10.1136/bjophthalmol-2020-316234 (PMC9132855; doi:10.1136/bjophthalmol-2020-316234)
Supplement: Supplementary data [file bjophthalmol-2020-316234supp002.pdf]

Supplementary Table 2: Cumulative incidence of high myopia according to spherical equivalent of refraction in diopters per age category.

**Age (years)**	**SER**	**Subjects (N)**	**Median survival time (Age, Y [95% CI])**	**5 years survival**	**10 years survival**
Under 10		*278*			
	≤ -0.5D to ≥ -1.5D	132	NR	95.6 (2.0)	85.8 (3.9)
	< -1.5D to ≥ -3.0D	120	24.9 (NE)	88.8 (3.6)	67.4 (5.4)
	< -3.0D to ≥ -4.5D	23	16.0 (12.9 – 19.0)	49.5 (12.3)	21.2 (10.7)
	< -4.5D to ≥ -6.0D	3	11.2 (10.0 – 12.5)	NR	NR
10 – 12		*751*			
	≤ -0.5D to ≥ -1.5D	285	NR	99.0 (0.7)	97.0 (1.7)
	< -1.5D to ≥ -3.0D	323	NR	96.9 (1.2)	85.8 (3.0)
	< -3.0D to ≥ -4.5D	106	24.4 (21.7 – 27.1)	80.9 (4.2)	55.6 (6.3)
	< -4.5D to ≥ -6.0D	37	16.2 (14.3 – 18.0)	40.7 (8.5)	17.2 (7.3)
13 – 15		*1083*			
	≤ -0.5D to ≥ -1.5D	371	NR	99.0 (1.0)	99.0 (1.0)
	< -1.5D to ≥ -3.0D	446	NR	100 (NE)	88.2 (6.0)
	< -3.0D to ≥ -4.5D	178	NR	94.4 (2.1)	76.8 (8.1)
	< -4.5D to ≥ -6.0D	88	19.9 (18.9 – 20.8)	48.1 (6.1)	7.6 (6.3)

95% CI = 95% confidence interval
NE = not evaluable
NR = not reached
